# Olive Leaf Extract Modulates Quorum Sensing Genes and Biofilm Formation in Multi-Drug Resistant *Pseudomonas aeruginosa*

**DOI:** 10.3390/antibiotics9090526

**Published:** 2020-08-19

**Authors:** Nazly R. El-sayed, Reham Samir, Lina Jamil M. Abdel-Hafez, Mohammed A. Ramadan

**Affiliations:** 1 Department of Microbiology and Immunology, Faculty of Pharmacy, 6 October University, Giza 12585, Egypt; Nazly.reda.Pha@o6u.edu.eg (N.R.E.-s.); Lina.jamil@ymail.com (L.J.M.A.-H.); 2 Department of Microbiology and Immunology, Faculty of Pharmacy, Cairo University, Cairo 11562, Egypt; mohamed.abdelhalim@pharma.cu.edu.eg

**Keywords:** *Pseudomonas*, biofilm, green tea, olive leaves, quorum sensing inhibitors, real-time RT-PCR

## Abstract

Biofilm acts as a complex barrier against antibiotics. In this study, we investigated the inhibitory activities of *Olea europaea* (olive) leaves *Camellia sinensis* (green tea), *Styrax benzoin*, *Ocimum basilicum*, *Humulus lupulus*, *Ruta graveolens*, and Propolis extracts on the biofilm formation, pyocyanin production, and twitching motility of *Pseudomonas aeruginosa* isolates. Moreover, we investigated the effect of olive leaf extract on the transcription of some biofilm related genes. A total of 204 isolates of *Pseudomonas* were collected from different Egyptian hospitals. A susceptibility test, carried out using the disc diffusion method, revealed that 49% of the isolates were multidrug-resistant. More than 90% of the isolates were biofilm-forming, of which 26% were strong biofilm producers. At subinhibitory concentrations, green tea and olive leaf extracts had the highest biofilm inhibitory effects with 84.8% and 82.2%, respectively. The expression levels of *lasI*, *lasR*, *rhlI*, and *rhlR* treated with these extracts were significantly reduced (*p* < 0.05) by around 97–99% compared to untreated isolates. This study suggests the ability of olive leaf extract to reduce the biofilm formation and virulence factor production of *P. aeruginosa* through the down regulation of quorum sensing (QS) genes. This may help in reducing our dependence on antibiotics and to handle biofilm-related infections of opportunistic pathogens more efficiently.

## 1. Introduction

*Pseudomonas aeruginosa* is an emerging opportunistic human pathogen primarily associated with hospital-acquired infection [1]. *P. aeruginosa* is implicated in the etiology of several diseases including bronchopneumonia, bacteremia, endocarditis, urinary tract infections, burns, and wound infections, etc. [2,3]. The prevalence of infections with *P. aeruginosa* is caused by the development of various extracellular products and cell-associated virulence factors, including pyocyanin, flagella, pili, and alginate [4,5]. Another factor which contributes to *P. aeruginosa* pathogenesis is its ability to form biofilms when attached to biotic and abiotic surfaces [6]. Biofilm is a complex population of microorganisms enclosed in a matrix [7]. The biofilm extracellular matrix consists of secreted extracellular polymeric substances made of exopolysaccharides (EPS), proteins, nucleic acids, and lipids, which serve as a barrier that keeps bacterial cells together [8]. The matrix is important because it provides structural stability, protects the biofilm, and retards the spread of antibiotics through the biofilm, thereby noticeably increasing the drug resistance among the microbial population [9,10].

Another virulence factor that *P. aeruginosa* produces is a blue-pigment called pyocyanin. It is a phenazine compound that generates reactive oxygen species [11]. Pyocyanin can easily attack biological membranes and impair cellular respiration by exhaustion at intracellular c-AMP and ATP levels. It is commonly recovered from cystic fibrosis patients infected with *P. aeruginosa* [4].

Among the cell-associated virulence factors are the cell surface structures, the type IV pili. They most likely promote the adherence to eukaryotic cells and abiotic surfaces [12]. They are also necessary for the flagellum-independent process of translocation which is called twitching motility. Type IV pilus-based twitching motility is sufficient to initiate attachment and production of *P. aeruginosa* biofilm [13].

Most bacteria regulate their virulence factors via a cell-to-cell signaling mechanism, called quorum sensing (QS). It requires the development of signaling molecules called autoinducers (AIs) [14]. These molecular signals spread throughout the community and interact with their corresponding regulators for collaborative expression of virulence specific genes such as extracellular proteases, efflux pumps, motility, attachment, and biofilm formation [15]. Acyl-homoserine lactones (AHLs) are the most important AIs in Gram-negative bacteria [16]. *P. aeruginosa* recruits at least four different QS networks interrelated to each other, namely *las*, *rhl*, *iqs*, and *pqs*. These systems possess transcriptional regulators LasR, RhlR, IqsR, and PqsR, respectively, which, after binding to their specific AIs, trigger the expression of selected genes linked to virulence [15] from which, several proteins that are engaged in pathogenicity and the development of biofilms [17]. In addition, transcriptomic data demonstrated the negative regulations of *P. aeruginosa* QS with laslI, laslR, rhlI, and rhlR under different conditions. Different studies proposed the possibility of the reversion of biofilm formation and how that would impact the progression of infectious diseases [18]. Several natural compounds have been tested experimentally for their ability to suppress the bacterial QS systems [19,20,21]. Unlike conventional antibiotics which affect certain cellular metabolic process, the quorum sensing inhibitors (QSIs) are believed to hinder bacterial communication without imposing pressure on the bacteria, thus reducing the probability for the emerging of resistant strains existence [22]. As an example of plant-derived compounds, catechin-7-xyloside, sappanol, and butein were capable of interacting with LasR and significantly reduced the biofilm formation, pyocyanin, elastase, and rhamnolipid without influencing the growth of *P. aeruginosa* [23].

The antimicrobial activity of some herbal medicines against different pathogens has been recorded from various countries [24]. Moreover, some natural products, primarily phytochemicals or their derivatives, may also have disturbing effects on quorum sensing (QS) systems [25,26]. Such phytochemicals are less harmful, have high chemical varieties, biochemical precision, and thus have an upper hand over traditional antibiotics [27]. 

As a new therapeutic approach: RhlR, one of the two acyl-homoserine-lactone circuits that regulate QS in *P. aeruginosa*, is considered to be a promising target to treat multidrug resistant *P. aeruginosa* [28]. RhlR is highly influenced by several small regulatory RNAs that adjust virulence factors regulated by QS in *P. aeruginosa* which are involved in shifting acute infections to chronic diseases [29]. 

The fast growing antibiotic resistance has directed attention toward nonantibiotic virulence inhibitors. This approach focuses on inhibiting virulence without killing the bacteria. Therefore, quorum sensing inhibitors were extensively studied. Numerous quorum sensing inhibitors have been identified against *P. aeruginosa* to help dealing with multi and pan drug resistant strains.

The objective of this research is to evaluate the impact of plant-derived natural products, as potential inhibitors, on biofilm formation, pyocyanin production, and twitching motility of multidrug resistant *Pseudomonas* isolates. In addition, transcriptional analysis of selected QS-controlled genes was performed after treatment with plant extracts. 

## 2. Results

### 2.1. Bacterial Isolates

Out of 332 isolates collected from various hospitals in Egypt between October 2016 and March 2018, a total of 91 clinical isolates and 113 environmental isolates were identified as *Pseudomonas aeruginosa*. A total of 43 (47%) clinical isolates were collected from urine samples, 25 (27%) from wounds, 13 (14%) from sputum samples, 4 (4%) from bronchoalveolar lavages, 3 (3%) from blood samples, 2 (2%) from throat swabs, and 1 (1%) from tracheal aspirate.

### 2.2. Antimicrobial Susceptibility Testing

Antimicrobial susceptibility screening was performed using the disc diffusion method according to the standard Clinical and Laboratory Standards Institute (CLSI), 2016. This study revealed that 49% of the tested isolates were multidrug-resistant (MDR). The tested isolates showed relatively high resistance to ticarcillin/clavulanic acid and cefepime with 54% and 50%, respectively. Additionally, relatively low resistance patterns were registered against colistin (7%) and tobramycin (18%), as shown in Table 1.

### 2.3. Biofilm Production

#### 2.3.1. Congo Red Assay

The biofilm formation ability of the tested isolates was primarily screened by Congo red assay, as shown in supplementary Figure S1. The Congo red method detected 187 isolates (92%) as biofilm producers. In this assay, we could not differentiate between moderate and weak biofilm-forming isolates (Table 2).

#### 2.3.2. Microtiter Plate Method

The results achieved from the microtiter plate method based on the measured optical denisty (OD) at 595 nm and compared to the negative control, demonstrated that 184 isolates (90%) were biofilm producers, while 20 isolates (10%) could not produce any detectable biofilm. This method was able to differentiate between strong, moderate, and weak biofilm-forming isolates, as shown in Table 2.

### 2.4. Phenotypic Detection of Other Virulence Factors

All isolates were tested and compared to twitching motility and pyocyanin production of standard *P. aeruginosa* (ATCC 12924), but only 85% showed positive twitching motility and 51.5% produced pyocyanin pigment.

It worth mentioning that two isolates (C21, E81) were indicated as both strong biofilm producers and of high antibiotic resistance, hence, these isolates were selected for further use in the subsequent experiments. 

### 2.5. Molecular Characterizations

The 16S rRNA gene sequence analysis of both tested isolates (C21, E81) matched with the sequence of the *Pseudomonas aeruginosa* strain ATCC 10145 16S ribosomal RNA partial sequence with percent identities of 99.05% and 98.99%, respectively.

### 2.6. MIC Determination, and Growth Curve Analysis of Selected Isolates

The MICs of seven plant extracts against selected *Pseudomonas* isolates were determined using the broth microdilution method in accordance with the CLSI 2016 recommendations [30], as shown in Table 3. The most effective plant extracts against the tested isolates were the *Camellia sinensis* (green tea) with MIC value 6.25 mg/mL, followed by *Olea europaea* (olive) extract with 12.5 mg/mL. 

Additionally, the growth curve analysis, as shown in Supplementary Figure S2, did not expose a significant difference (*p* > 0.05) in the growth of *P. aeruginosa* C21 and E81 isolates treated with sub-MIC (½ MIC) of each of the tested herbal extracts compared with the growth of the untreated bacterial cultures.

### 2.7. Effect of Sub-Inhibitory Concentrations of Tested Extracts on Biofilm Formation

**Figure 1:**
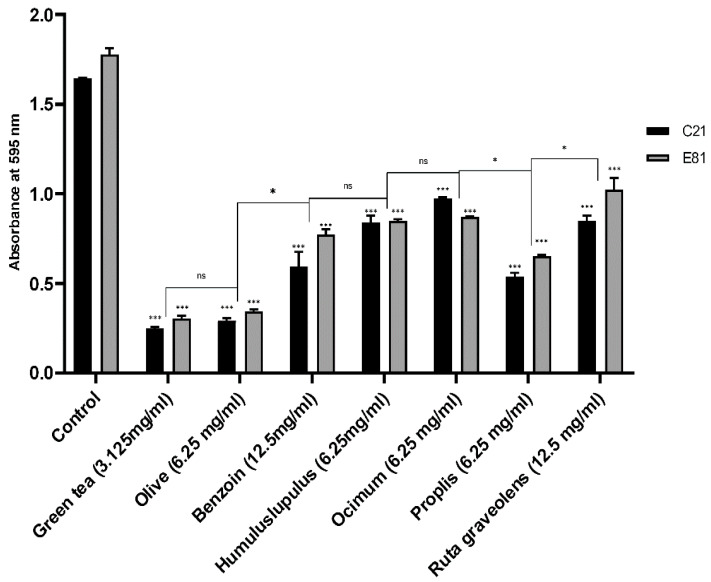
Effect of different herbal extracts on biofilm formation of *P. aeruginosa*. Bar chart showing the absorbance of *P. aeruginosa* C21 and E81 in absence and presence of the sub-MIC of *Camellia sinensis* (3.125 mg/mL), *Olea europaea* (6.25 mg/mL), *Styrax benzoin* (12.5 mg/mL), *Humulus lupulus* (6.25 mg/mL), *Ocimum basilicum* (6.25 mg/mL), Propolis (6.25 mg/mL), and *Ruta graveolens* (12.5 mg/mL). Biofilm formation was evaluated using the microtiter plate method. Data are an average of three independent experiments. The results are presented by the mean ± SD. ns = nonsignificant; * = *p* < 0.05; *** = *p* < 0.001: significance was compared to the respective control.

Biofilm formation, of the clinical (C21) and environmental (E81) *P. aeruginosa* isoaltes, was evaluated in the absence (negative control) and the presence of tested herbal extracts (½ MIC) in a 96-well microtiter using the Microtiter plate method. The sub-MICs (½ MIC) of *Camellia sinensis*, *Olea europaea*, *Styrax benzoin*, *Humulus lupulus*, *Ocimum basilicum*, Propolis, and *Ruta graveolens* extracts inhibited the biofilm formation of the clinical (C21) isolate by 84.8%, 82.2%, 63.7%, 48.8%, 40.7%, 67.2%, and 48.3%, respectively. However, the same extracts inhibited the biofilm formation of the environmental *P. aeruginosa* isolate (E81) by 82.7%, 80.6%, 56.3%, 52%, 50.6%, 63.1%, and 42.2%, respectively, compared to the untreated control. Both isolates exhibited significant inhibition in biofilm formation when treated by the sub-MIC of all herbal extracts. Green tea and olive leaf extracts showed the highest inhibitory effects when compared to other plant extracts, as illustrated in Figure 1.

### 2.8. Effect of Sub-Inhibitory Concentrations of Tested Extracts on Twitching Motility

**Figure 2:**
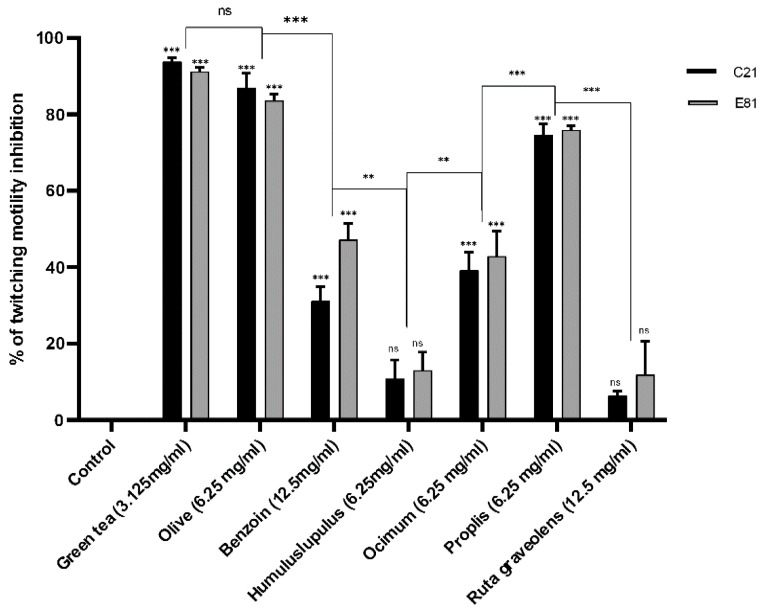
Effect of different herbal extracts on twitching motility of *P. aeruginosa*. Bar chart showing the percent inhibition of C21 and E81 twitching motility in the absence and presence of herbal extracts *Camellia sinensis* (3.125 mg/mL), *Olea europaea* (6.25 mg/mL), *Styrax benzoin* (12.5 mg/mL), *Humulus lupulus* (6.25 mg/mL), *Ocimum basilicum* (6.25 mg/mL), Propolis (6.25 mg/mL), and *Ruta graveolens* (12.5 mg/mL). Data are an average of three independent experiments. The results are presented by the mean ± SD. ns = nonsignificant; ** = *p* < 0.01; *** = *p* < 0.001: significance was compared to the respective control.

Twitching motility of the clinical isolate C21 and the environmental isolate E81 was determined in the absence (negative control) and the presence of tested herbal extracts (½ MIC). The sub-MIC (½ MIC) of *Camellia sinensis*, *Olea europaea*, *Styrax benzoin*, *Humulus lupulus*, *Ocimum basilicum*, Propolis, and *Ruta graveolens* extracts inhibited the development of twitching motility in the clinical isolate (C21) by 93.8%, 31.2%, 87%, 10.8%, 39.3%, 74.6%, and 6.5%, respectively. However, the sub-MIC of the same extracts inhibited the development of twitching motility in the environmental isolate(E81) by 91.3%, 47.3%, 83.6%, 12.9%, 42.9%, 75.9%, and 11.9%, respectively, compared to the untreated control. Both isolates showed no significant inhibition in twitching motility when treated by the sub-MIC of *Humulus lupulus* and *Ruta graveolens* extracts and significantly inhibited when treated with the sub-MIC of *Camellia sinensis*, *Olea europaea*, *Styrax benzoin*, *Ocimum basilicum*, and Propolis extracts. Green tea and olive leaf extracts showed the highest inhibitory effects when compared to other plant extracts, as illustrated in Figure 2.

### 2.9. Effect of Sub-Inhibitory Concentrations of Tested Extracts on Pyocyanin Production

**Figure 3:**
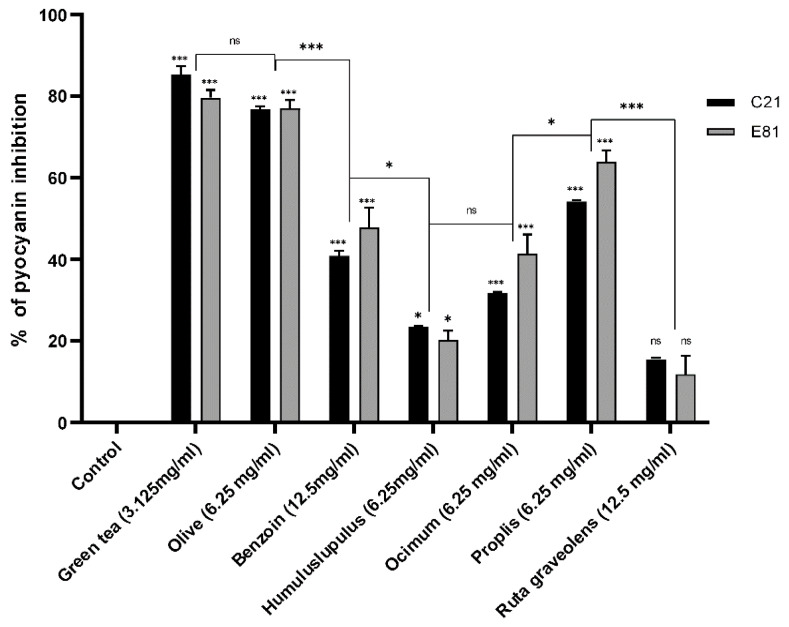
Effect of different herbal extracts on pyocyanin production of *P. aeruginosa*. Bar chart showing percent inhibition of C21 and E81 pyocyanin production in the absence and presence of herbal extracts *Camellia sinensis* (3.125 mg/mL), *Olea europaea* (6.25 mg/mL), *Styrax benzoin* (12.5 mg/mL), *Humulus lupulus* (6.25 mg/mL), *Ocimum basilicum* (6.25 mg/mL), Propolis (6.25 mg/mL), and *Ruta graveolens* (12.5 mg/mL). Pyocyanin production was evaluated by the chloroform-HCl extraction method. Data are an average of three independent experiments. The results are presented by the mean ± SD. ns = nonsignificant; * = *p* < 0.05; *** = *p* < 0.001: significance was compared to the respective control.

Pyocyanin production of the clinical isolate C21 and the environmental isolate E81 were determined in the absence (negative control) and the presence of tested herbal extracts (½ MIC) by the chloroform-HCl extraction method. The sub-MIC (½ MIC) of *Camellia sinensis*, *Olea europaea*, *Styrax benzoin*, *Humulus lupulus*, *Ocimum basilicum*, Propolis, and *Ruta graveolens* extracts inhibited the pyocyanin production of the clinical isolate (C21) by 85.4%, 40.8%, 76.8%, 23.5%, 31.8%, 54.2%, and 15.6%, respectively. However, the sub-MIC (½ MIC) of the same extracts inhibited the pyocyanin production of the environmental isolate (E81) by 79.8%, 47.6%, 77.1%, 20.3%, 41.5%, 63.9%, and 11.8%, respectively, compared to the control. Both isolates showed no significant inhibition in pyocyanin production when treated by the sub-MIC of *Ruta graveolens* extract and significantly inhibited when treated by the sub-MIC of *Camellia sinensis*, *Olea europaea*, *Styrax benzoin*, *Humulus lupulus*, *Ocimum basilicum*, Propolis, and *Ruta graveolens* extracts. Green tea and olive leaf extracts showed the highest inhibitory effects when compared to other plant extracts (Figure 3).

These results have motivated us to study the impact of green tea and olive leaf extracts on the gene transcription of QS systems (*lasI*, *lasR*, *rhlI*, and *rhlR*).

### 2.10. Effect of Camellia Sinensis and Olea Europaea Leaf Extracts on the QS Genes Transcription Levels of P. aeruginosa C21

**Figure 4:**
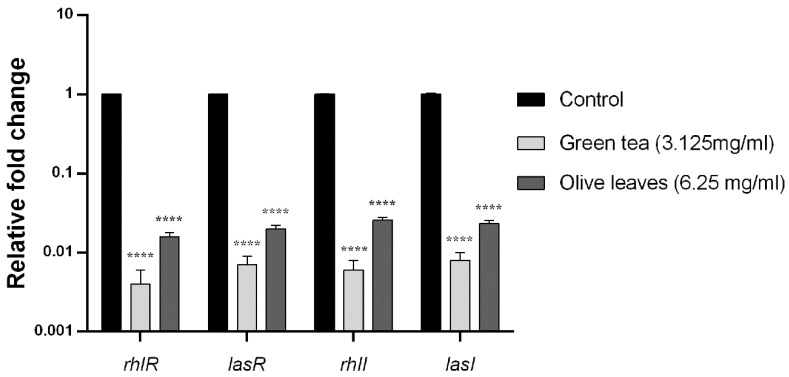
Effect of *Camellia sinensis* and *Olea europaea* extracts on the transcription of quorum sensing (QS)-regulated genes of *P. aeruginosa*C21. Bar chart of the qRT-PCR analysis representing the relative transcription levels of *lasI*, *lasR*, *rhlI*, and *rhlR* genes treated with sub-MIC levels of *Camellia sinensis* (3.125 mg/mL) and *Olea europaea* (6.25 mg/mL), normalized with the reference gene *5s RNA* and compared to the untreated control. The error bars indicate the standard deviations of three replicates. **** = *p* < 0.0001: significance was compared to the respective control.

The influence of the *Camellia sinensis* and *Olea europaea* herbal extracts on QS genes transcription was evaluated by fluorescence real-time PCR. The fold change and the percent of inhibition of *lasI*, *lasR*, *rhlI*, and *rhlR* genes of *Camellia sinensis* extract-treated clinical isolate (C21) were 99.6%, 99.3% 99.4%, and 99.2%, respectively. Moreover, the fold change of the same genes of *Olea europaea* extract-treated clinical isolate (C21) was 98.4%, 98%, 97.4, and 97.7%, respectively, compared to the housekeeping gene *5s RNA* (Figure 4).

### 2.11. Effect of Camellia Sinensis and Olea Europaea Leaf Extracts on the QS Genes Transcription Levels of P. aeruginosa E81

**Figure 5:**
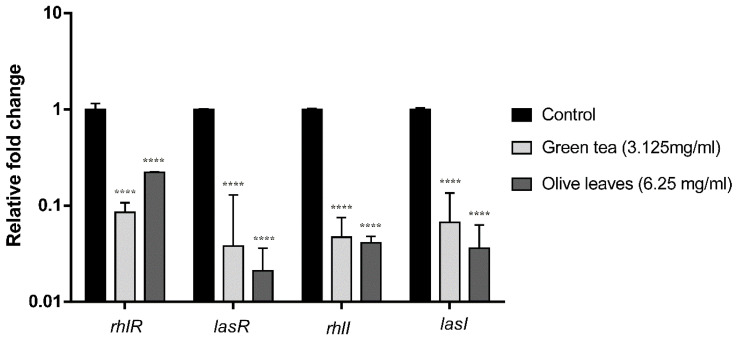
Effect of *Camellia sinensis* and *Olea europaea* extracts on the expression of QS-regulated genes of *P. aeruginosa* (E81). Bar chart of the qRT-PCR analysis representing the relative transcription levels of *lasI*, *lasR*, *rhlI*, and *rhlR* genes treated with sub-MIC levels of *Camellia sinensis* (3.125 mg/mL) and *Olea europaea* (6.25 mg/mL), normalized with the reference gene *5s RNA* and compared to the untreated control. The error bars indicate the standard deviations of three replicates. **** = *p* < 0.0001: significance was compared to the respective control.

The fold change and the percent of inhibition of *lasI*, *lasR*, *rhlI*, and *rhlR* genes transcription of *Camellia sinensis* extract-treated environmental isolate (E81) were 91.5%, 96.2%, 95.3%, 95.3%, and 93.3%, respectively. Additionally, the fold change of the same genes of *Olea europaea* extract-treated environmental isolate (E81) was 77.9%, 97.9%, 95.9%, 96.4%, and 93.3%, respectively as shown in Figure 5.

## 3. Discussion

*Pseudomonas aeruginosa* is the most prevalent opportunistic pathogen which can lead to nosocomial infections in patients who are immune-compromised or suffer from cystic fibrosis [31]. The resistance of *P. aeruginosa* is causing a devastating problem and limiting therapeutic choices. Moreover, the pathogenicity of this organism is high, due to the production of a set of well-regulated virulence factors.

In our study, the highest resistance was recorded against ticarcillin/clavulanic acid, cefepime, and levofloxacin with 54%, 50%, and 47%, respectively. *P. aeruginosa* possesses numerous intrinsic and acquired resistance mechanisms that are used to overcome most conventional antibiotics and to adapt to newly applied antimicrobial agents. Our findings were consistent with previous studies which observed that the inappropriate use of antibiotics during treatment enhanced the production of MDR- *P. aeruginosa* isolates, leading to the ineffectiveness of the empiric antibiotic therapy towards this bacterium [32,33]. Similar studies on the incidence of resistance to different antibiotics were also reported [34]. 

On the other hand, the most effective antibiotics against *P. aeruginosa* were colistin with a susceptibility rate of 93% followed by tobramycin, meropenem, gentamycin, amikacin, and imipenem with 81%, 72%, 72%, 69%, and 68%, respectively. Similar results were recorded by Mustafa and coworkers, as colistin was the most effective antibiotic against *P. aeruginosa* isolates with 92% susceptibility followed by tobramycin, meropenem, and imipenem 72%, 63%, and 48%, respectively [35]. A previous study conducted in Egypt reported a rise in the numbers of imipenem tolerant *P. aeruginosa* [36]. Moreover, another study showed that the lowest antibiotic resistance was to imipenem with 7%, while the highest resistance was to amikacin—48% [37]. In another study in Egypt, it was found that most of the *P. aeruginosa* isolates were resistant to ceftazidime followed by levofloxacin and imipenem but with a lower resistance to amikacin [38]. The variation in the resistance rates among previous studies can be due to the differences in geographical distribution, time of sample collection, number of isolates gathered in each study, and the difference in antibiotic strategies adopted in every region.

In our study, the results revealed that about 90% of the tested isolates (204 isolates) were biofilm producers. The microtiter plate method was considered as the gold standard for this study and was compared with data from the Congo red method. The Congo red method, as a subjective way of testing biofilm formation, was less precise than microtiter plate method, but, on the other hand, it was quicker and required fewer materials for the detection of biofilm formation [39]. Our isolates were categorized according to their biofilm formation ability into 26% strong biofilm producers, 39% moderate biofilm producers, 25% weak biofilm producers, and 10% could not form any detectable biofilm. Many studies showed differences in the levels of biofilm production by *P. aeruginosa* isolates [40]. For example, Abdelraheem et al., found that biofilm formation was observed in 27% *Pseudomonas* clinical isolates; 14% developed strong biofilm, 7% developed moderate biofilm, and 6% developed weak biofilm [37]. A different study in Egypt demonstrated that biofilm formation was observed in 91.4% *P. aeruginosa* isolates; 25.7%, 40%, 25.7%, and 8.6% of isolates were strong, moderate, weak, and nonbiofilm producers, respectively [38]. The variation in findings between different studies can be related to several variables, including the differences in type and number of strains gathered in each research study, that eventually lead to differences in the ability of isolates to produce biofilms. In this report, MDR strains were higher among biofilm-producers—with 48.4% (89/184)—than nonbiofilm producing *P. aeruginosa isolates* 30% (6/20). A different study with similar findings noticed that MDR isolates occurred in both biofilm formers and nonbiofilm formers, but most of them were considerably related to the biofilm formers [41]. This may be due to the reduction in growth rates of the bacteria in the biofilm [42], over expression of efflux pump [43], and accelerated expression of resistance genes among bacteria inside the formed matrix [44,45]. Besides, this matrix has a protective effect that hinders the penetration of antibiotics into the bacterial cells embedded in the biofilm community [37,46].

From here, the need for new therapeutics that can hinder the biofilm formation and decrease the virulence of *P. aeruginosa* without inducing more resistance is continuously growing. Plant derived products are always a good option due to their effectiveness and considerably low side effects. Numerous classes of molecules from natural sources were reported to have both antibiofilm formation and anti-QS properties. We selected *Olea europaea* (olive) leaves *Camellia sinensis* (green tea), *Styrax benzoin*, *Ocimum basilicum*, *Humulus lupulus*, *Ruta graveolens*, and Propolis because they have multiple use in folk medicine as antimicrobial agents or as treatments for wound infections or bed ulcers.

All of the tested plant extracts showed variable antibacterial and antivirulence activities against *P. aeruginosa* isolates. On the other hand, *Camellia sinensis* and *Olea europaea* were the most potent antibacterial agents. In addition, their sub-MIC efficiently inhibited biofilm formation and virulence factor production by *P. aeruginosa* and significantly down regulated *lasI*, *lasR*, *rhlI*, and *rhlR* expression without affecting cell growth. We can relate these effects to their rich constituents of bioactive compounds (Table S2). 

The activities of green tea are mainly due to the presence of polyphenols, the most abundant of which is Catechin, particularly epigallocatechin gallate (EGCG) which is the most effective catechin representing 50%–80% of the total catechin content and is known for its inhibitory effect on *P. aeruginosa* PAO1 virulence factors [47]. Apparently, the observed antibiofilm activity of *olea europaea* is attributed to the high concentrations of phenolic compounds, such as oleuropein and hydroxy tyrosol [48], or maybe due to the synergistic effect of some phenols contained in the olive extracts. Moreover, phenolic compounds can, potentially, increase the permeability of cell membranes, thus facilitating their rupture [49]. The obtained results also agreed with Yin et al.; they reported that tea polyphenol (TP) exhibited potent antimicrobial activity on all selected isolates and it can also inhibit the development of total protease, elastase, pyocyanin, biofilm formation, and swarming motility in *P. aeruginosa* [50]. Another report by Qais et al. revealed that the sub-MICs of green tea ethyl acetate fraction (GTEF) inhibited numerous virulence factors of *P. aeruginosa* PAO1 pyocyanin, pyoverdine, protease, elastase, rhamnolipid production, and swimming motility. Additionally, GTEF demonstrated wide-spectrum antibiofilm action with a more than 80% reduction in the biofilm formation of the tested organisms [51]. Tea catechin also inhibited the production of violacein in *C. violaceum* and virulence factors in *Pseudomonas aeruginosa* PAO1 [52]. Epigallocatechin gallate (EGCG) was found to be the most powerful catechin [47]. In addition, a study conducted by Najee et al. showed that *Olea europaea* fatty oil inhibited the formation of biofilm on drug-resistant bacterial and fungal pathogens by interacting efficiently with microbial surface structures necessary for growth, such as membrane, and also indicated that it was capable of reducing the motility of the pathogen [53]. Another study showed that the methanolic extract of olive leaves demonstrated essential antioxidant and antibiofilm activities [54]. Additionally, olive leaf is reported to be a potentially inexpensive, sustainable, and plentiful origin of biophenols [55]. Our findings demonstrated that the difference between both tested extracts may be due to their differences in the type and concentration of the active constituents, as well as the polyphenol contents (2.93 and 2.07 mg/kg, respectively). The Phytochemical screening for green tea and olive revealed the presence of alkaloids, flavonoids, tannins, saponins, glycoside, terpenoids, and anthocyanin. These results are consistent with other studies that reported the same discovered data [56,57]. These compounds are considered to be bioactive and may be responsible for the activities of both extracts. The down regulation in *lasI*, *lasR*, *rhlI*, and *rhlR* genes can be due to their binding affinity to QS regulatory proteins by competitively reducing the attachment of natural autoinducers (AHL). 

Many studies examined the effect of other natural sources as anti-QS and antibiofilm on *P. aeruginosa*. Between them, plant-derived natural flavonoid and phenolic compounds are widely studied for the suppression of *P. aeruginosa* biofilm [58] and QS regulated virulence factors [59]. Flavonoids such as catechin were observed to suppress the expression of QS genes, and thereby reduce virulence factors in *P. aeruginosa* PAO1 [52]. Subsequently, other functionally-related flavonols, such as baicalein [60,61], and quercetin [19], are also anti-QS and anti-biofilm compounds.

## 4. Conclusions

Our study showed that green tea and olive leaf extracts were exhibited to be the most efficient plant extracts against planktonic and biofilm cells. To our knowledge, we report for the first time the ability of olive leaf extract to reduce the biofilm formation of *P. aeruginosa* and virulence factor production through the down regulation of QS genes. Moreover, olive (*Olea europaea*) leaf is considered as a cheap, abundant, and renewable source of biophenols. These findings may help in reducing our dependence on antibiotics and to handle biofilm-related infections of opportunistic pathogens more efficiently. Further studies are required to distinguish the most important phytochemical compounds and estimate their antibiofilm activities and their mechanisms of action. In addition, in vivo studies are required to enable their application in the prevention and treatment of biofilm-related *P. aeruginosa* infections.

## 5. Materials and Methods

### 5.1. Sample Collection and Growth Conditions

Throughout the period from October 2016 to March 2018, a total of 332 bacterial cultures were recovered from Qasr El-Aini, October 6 University Hospital, Dar El Salam Cancer Center, Al-Helal hospital, National Cardiology Institute, EL-Nile Badrawy hospital, Abu Al-Reish hospital, Sheikh-Zayed Specialized hospital, National Cancer Institute, and Medical Research Institute-Alexandria, Egypt. These bacterial isolates were recovered from different clinical specimens of hospitalized patients after obtaining informed consents. Clinical specimens were collected from the wound, burn, urine, and sputum. Environmental samples were collected from samples of water and fomites in hospital settings. 

Fresh samples were cultivated on Cetrimide agar media, after which isolated species were identified by a regular microbiological technique such as colony morphology (green exopigment Cetrimide agar), Gram staining (Gram-negative rods), and biochemical reactions (oxidase-positive and citrate-positive). Identification at the species level was performed by using the Microbact™ Gram-negative system. The Microbact system was implemented in compliance with the manufacturer’s protocol (Oxoid, UK). *Pseudomonas aeruginosa* (ATCC 12924) standard strain was used as a positive control.

### 5.2. Antimicrobial Susceptibility Testing

Antibiotic susceptibility patterns of 204 *Pseudomonas* spp. identified isolates that were examined by the Kirby Bauer Disc Diffusion method depending on the protocol described by the clinical and laboratory standard institute 2016 [30]. Using a sterile loop, 3–5 colonies were picked from each bacterial isolate, and each colony was added to 5mL of sterile 0.9% saline to prepare a standardized 0.5 McFarland bacterial suspension. Sterilized Mueller-Hinton agar (MHA) plates were consistently inoculated with the bacterial suspension using sterile swabs. Antibiotic discs were transferred to the plates’ surfaces, and the plates were then incubated at 37 °C for 24 h. The diameters of the antibiotic inhibition zone were measured after incubation. 

Antibiotics were selected, and antimicrobial susceptibility results were interpreted in compliance with recommendations of the CLSI guidelines [30] (Table S1). Antibiotic discs (Hi-media, UK) used in this study were amikacin (30 µg), aztreonam (30 µg), cefepime (30 µg), ceftazidime (30 µg), ciprofloxacin (5 µg), colistin (10 µg), gentamicin (10 µg), imipenem (10 µg), levofloxacin (5 µg), meropenem (10 µg), piperacillin + tazobactam (100/10 µg), ticarcillin/clavulanic acid (10/75 µg), and tobramycin (10 µg).

### 5.3. Biofilm Formation Detection Methods

Two phenotypic methods were used to test the biofilm-forming ability of the *Pseudomonas* spp. Isolates, namely the Congo red agar method and the Microtiter plate method.

#### 5.3.1. Congo Red Agar Method

The Congo red agar medium consisted of brain heart infusion broth (BHI) (37 gm), agar (10 gm), sucrose (5 gm), and Congo red stain (0.8 gm) per liter of distilled water [62]. Congo red dye was individually prepared and sterilized. After cooling, the dye was eventually added to the sterile BHI agar medium enhanced with sucrose. Plates were inoculated with the tested isolates and incubated at 37 °C for 24 h. The color of the bacterial colonies was an indication of exopolysaccharide (EPS) production. Isolates with multiple black colonies have been categorized as positive for biofilm production, whereas red and pink colonies have been classified as negative [63]. 

#### 5.3.2. 96 Well Microtiter Plates Method

The in vitro formation of biofilms in 96 well microtiter plates containing Luria Bertani (LB) broth medium was evaluated as follows [64]: Briefly, the overnight broth cultures of the isolated bacterial strains were calibrated, approximately, to 0.5 McFarland turbidity standard, diluted to 1:100 in LB broth, and inoculated into a microtiter plate (200 μL per well). The microtiter plate was incubated at 37 °C for 24 h. Negative control wells were also included (uninoculated broth). After incubation, the planktonic cells were withdrawn, and were rinsed three times with 200 μL of phosphate buffer saline. A volume of 200 μL of 0.1% of crystal violet (CV) was added to each well for 15 min at ambient temperature. The excessive dye was washed away by soaking in distilled water. A volume of 200 μL of 33% acetic acid was added to each well for 10–15 min to solubilize the CV. The optical density (OD) was calculated at 595 nm in a plate reader using acetic acid as a blank. Each growing strain was tested three times, and the mean was obtained. Then, the assessment of biofilm production was categorized according to the criteria of Stepanovic et al. as follows: ODc was defined as three standard deviations (SDc) above the mean OD of the negative control. Furthermore, the isolates were classified according to resulted OD into categories: nonbiofilm producer (OD ≤ ODc), week biofilm producer (OD ≤ 2ODc), moderate biofilm producer (2ODc < OD ≤ 4ODc), or strong biofilm producer (OD > 4ODc) [64].

### 5.4. Pyocyanin Assay

Pyocyanin pigment development for the *Pseudomonas* spp. isolates was evaluated by using King A agar. Slants of the medium were inoculated and incubated for four days at 30–32 °C. Pyocyanin production was revealed by the addition of 0.5–1 mL chloroform to the inoculated slants, which were shaken for a few minutes until the pyocyanin was diffused, rendering the solvent blue. After that, the chloroform was acidified with a few drops of HCl, which resulted in a sudden shift in color from blue to red; this shift in color indicated the existence of pyocyanin.

For the quantification of the pyocyanin production, overnight culture was diluted with a ratio of 1:100 with fresh LB broth. Then, 25 mL of the fresh culture was grown at 37 °C, 200 rpm for 24 h. Afterwards, the supernatant was collected after centrifugation at 10,000 rpm for 10 min; then, a volume of 4.5 mL of chloroform was added to 7.5 mL of supernatant and vortexed. After the color of chloroform turned to green-blue, the mixture was centrifuged for 10 min at 10000 rpm. A volume of 3 mL of the resulting blue layer at the bottom (chloroform + pyocyanin) was transferred to a new tube. Subsequently, a volume of 1.5 mL of 0.2 M HCl was added to each tube and vortexed. After turning of the blue color into pink (top layer), the mixture was centrifuged for 2 min at 10,000 rpm, and then 1 mL from the pink layer was transferred to cuvettes, and the absorbance was measured at 520 nm spectrophotometrically. Finally, pyocyanin concentration (µl/mL) was calculated by multiplying the OD at 520 nm with 17.072 × 1.5. All centrifugation steps took place at 4 °C. An amount of 0.2 M HCl was used as a blank in spectrophotometry [65].

### 5.5. Twitching Motility Assay

A colony of each tested isolate was inoculated deep into the Luria Bertani (LB) agar plate with a sterile toothpick up to the bottom of the petri dish and incubated at 25 °C for 48 h. The appearance of a hazy growth area was noticed and measured at the surface between the agar and petri dishes [66]. The agar plates were gently removed, rinsed with water to eliminate any unattached cells, and then stained with 0.1% crystal violet for 1 min to see the twitching motility [67].

### 5.6. Molecular Identification

Two isolates (a clinical (C21) and an environmental (E81) isolate) with the highest antibiotic-resistance and the strongest biofilm production were further identified by 16S rRNA amplification and sequencing as follows: Overnight broth cultures of the isolated strains were centrifuged for 15 min at ambient temperature. The supernatants were eliminated, and the pellets were utilized for bacterial DNA extraction using the GeneJET^TM^ PCR Purification kit (Thermo Scientific) according to the manufacturer’s instructions. The dignity of the nucleic acids was evaluated on 1% agarose gels electrophoresis containing ethidium bromide, and DNA was stored at −20 °C. PCR amplification using the universal primers 16S-27F (5′AGAGTTTGATCCTGGCTCAG 3′) and 16S-1492R (5′TACGGTTACCTTGTTACGACTT 3′) was carried out in a thermal cycler (ABI, Applied Biosystems). Each PCR reaction was conducted using 25 µL of Maxima^®^ Hot Start PCR Master Mix (2X), 0.4 µL of each primer (10 µM), 5 µL of template DNA, and 18 µL nuclease-free water. Reaction without DNA was utilized as a negative control. The 16S rRNA gene sequences were compared with the NCBI GenBank database by the Blastn tool in order to identify the two isolates. The two 16S rRNA sequences were submitted to the NCBI database with accession numbers MT772093 and MT772097 for C21and E81, respectively.

### 5.7. Phytochemical Studies

#### 5.7.1. Plant Material

A total of seven medicinal plants listed in Table 4 were collected from the Experimental Station of Medicinal and Aromatic Plants, Faculty of Pharmacy, Cairo University, Giza, Egypt in August 2018. The taxonomical features were kindly confirmed by Dr. Abdel-Halem Abdel-mogali, a specialized taxonomist at the agriculture research center, Giza. The plants were air-dried, powdered, and kept tightly closed in glass containers until being extracted.

#### 5.7.2. Preparation and Characterization of Herbal Extracts

The dried powdered samples of the plants (200–300 g) were extracted with 70% ethanol by maceration several times until the plant materials were completely exhausted. The ethanolic solutions were filtered and then concentrated using a rotary evaporator under reduced pressure below 50 °C. The extracts were totally dried to obtain semisolid residues. The dried alcoholic plant extracts were stored at −20 °C in sealed, tightly closed glass containers. For both *Camellia sinensis* and *Olea europaea* alcoholic extracts, qualitative phytochemical analysis according to Savithramma et al. [68] and polyphenol contents using the Folin–Ciocalteu method [69] were carried out.

#### 5.7.3. Detection of MIC by the Broth Microdilution Method

The minimum inhibitory concentrations (MICs) of the active extracts were evaluated against strong-biofilm forming *Pseudomonas* species only using the broth microdilution method [70]. The Stock solutions of plant extracts were prepared as 100 mg/mL in dimethyl sulfoxide (DMSO) (twice the required concentration). Serial two-fold dilutions were prepared in Muller Hinton Broth medium (MHB) from 50–0.097 mg/mL of the plant extracts, and then, an inoculum of bacterial isolates was prepared in BHI broth calibrated to 0.5 McFarland. The experiment was performed in flat-bottom sterile 96-well microtiter plates—initial dispensation of 100 μL of bacteria and 100 μL of specific concentration of each plant extract. This 96 well plate was further incubated at 37 °C for 24 h. Each plant extract was investigated for each concentration in triplicates, and the experiment was performed three times independently.

#### 5.7.4. Growth Curve Analysis

Growth curve analysis was carried out to evaluate the effect of the sub-MIC of herbal extracts on the growth rates of selected isolates used in this study (*P. aeruginosa* C21and E81). Overnight cultures of the tested strains were inoculated into 100 mL of LB broth; OD was set to 0.5 McFarland. Each strain was grown in the presence of sub inhibitory concentrations (1/2 MIC) of each herbal extract concurrent with untreated inoculum as a control. The flasks were incubated at 37 °C, and OD600 was observed at 2 h intervals for up to 24 h.

#### 5.7.5. Effect of Subinhibitory Concentrations on Biofilm Formation

Antibiofilm activity of each plant extract against selected *Pseudomonas* isolates (C21 and E81) was evaluated at the sub-MIC concentrations (1/2 MIC) using a polystyrene microtiter plate method, as mentioned before, but with some modifications [71]. The sub-MICs amount of each plant extracts was added to the wells; extracts-free wells were used as controls. The percentage of biofilm inhibition was calculated using the following formula:

$$\%\;\mathrm{Inhibition}=\frac{\mathrm{OD}\;\mathrm{control}\;-\mathrm{OD}\;\mathrm{sample}}{\mathrm{OD}\;\mathrm{control}}\times 100$$

#### 5.7.6. Effect of Subinhibitory Concentrations on Twitching Motility

The tested isolates (C21 and E81) were cultured by stabbing into LB agar plates complemented by the sub-MIC (1/2 MIC) of each plant extract using sterile toothpicks, incubated overnight at 37 °C, and then remained for one to two days at room temperature (<25 °C). The diameter of the stained zone (mm) was measured to test the twitching motility; the experiment was carried out in triplicates. 

#### 5.7.7. Effect of Subinhibitory Concentrations on Pyocyanin Production

The influence of herbal extracts on pyocyanin production by *Pseudomonas* isolates (C21 and E81) was assayed using a colorimetric method as described before [72]. *Pseudomonas* isolates (100 μL, 10^6^ CFU/mL) were inoculated into fresh LB broth and were cultivated in the presence or absence of the sub-MICs (1/2 MIC) of plant extracts incubated at 37 °C for 24 h. Then, bacterial cultures were centrifuged at 4 °C, and the supernatant was gathered. Chloroform was added to the supernatant, and the mixture was shaken well. The chloroform layer was acidified by 0.2 M HCl to obtain the pink layer. Then, the absorbance of this layer was measured at 520 nm. Production of pyocyanin was determined as a ratio between the absorbance treated and the absorbance of the control [73]. The most efficient two plant extracts in biofilm formation were selected for the subsequent studies.

$$\%\;\mathrm{Inhibition}=\frac{\mathrm{Abs}\;\mathrm{control}-\mathrm{Abs}\;\mathrm{sample}}{\mathrm{Abs}\;\mathrm{control}}\times 100$$

### 5.8. Expression of the QS Genes

Fluorescence real-time PCR was used to monitor the expression of the QS genes of C21 and E81 in the presence and absence of the selected plant extracts. The primers that were utilized to amplify *lasI*, *lasR*, *rhlI*, *rhlR*, and 5S RNA (housekeeping gene) were demonstrated in Table 5. Total RNA was extracted in compliance with the manufacturer’s instructions of the Quick-RNA^TM^ MiniPrep extraction kit (Zymo research CORP, Australian). Reverse transcription of the total RNA was performed using a High-Capacity cDNA Reverse Transcription Kit (Thermo-Fischer Scientific). Real-time PCR was conducted using SYBR green kits (SensiFAST SYBR No-ROX Kit, Meridian Life science, UK). The reaction conditions for these genes were as follows: 95 °C for 15 min followed by 40 cycles of 95 °C for 15 s, 60 °C for 30 s, and 72 °C for 30 s [19]. The relative fold changes of mRNA levels were measured using the comparative cycle threshold (ΔΔCt) method for gene expression. The fold change in gene expression was standardized to the reference gene (*5S RNA*).

### 5.9. Statistical Analysis

The results are demonstrated as the mean values and standard deviations of three separate experiments. All statistical analyses of the variances between the controls and tests were conducted using one-way ANOVA followed by a posthoc Tukey test by GraphPad Prism 8 program, and only results at *p* < 0.05 were regarded as significant.

## Figures and Tables

**Table 1:** Antimicrobial susceptibility of the tested isolates against respective antibiotics.

Antimicrobial Agent	Number (%)		
	Susceptible (s)	Intermediate (I)	Resistant (R)
Amikacin (AK)	141 (69)	22 (11)	41 (20)
Aztreonam (AT)	109 (53)	55 (27)	40 (20)
Cefepime (CPM)	56 (27)	46 (23)	102 (50)
Ceftazidime (CAZ)	114 (56)	22 (11)	68 (33)
Ciprofloxacin (CIP)	130 (64)	17 (8)	57 (28)
Colistin (CL)	189 (93)	-	15 (7)
Gentamicin (GEN)	147 (72)	6 (3)	51 (25)
Imipenem (IPM)	140 (68)	22 (11)	42 (21)
Levofloxacin (LE)	133 (65)	17 (8)	54 (47)
Meropenem (MRP)	148 (72)	12 (6)	44 (22)
Piperacillin/Tazobactam (PIT)	85 (42)	64 (31)	55 (27)
Ticarcillin/Clavulanic acid (TCC)	47 (23)	46 (23)	111 (54)
Tobramycin (TOB)	165 (81)	3 (1)	36 (18)

**Table 2:** Screening of 204 isolates for biofilm formation by MTP and CRA methods.

Screening Method	Biofilm Formation N (%)				
	Strong	Moderate	Week	Non	*P. aeruginosa* (ATCC 12924)
MTP	54 (26%)	80 (39%)	50 (25%)	20 (10%)	OD 595 (1.03 ± 0.035)
CRA	52 (25%)	135 (66%)		16 (8%)	Intermediate

MTP: Microtiter plate method, CRA: congo red agar method, N: number of isolates. Experiments were performed in triplicate and values expressed as the mean ± standard deviation.

**Table 3:** Determination of MICs of plant extracts against the selected strong biofilm producer isolates.

Isolate	MICs of Different Plant Extracts (mg/mL)						
	*Camellia sinensis*	*Styrax benzoin*	*Olea europaea*	*Humulus lupulus*	*Ocimum basilicum*	*Propolis*	*Ruta graveolens*
C21	6.25	25	12.5	12.5	12.5	12.5	25
E81	6.25	25	12.5	12.5	12.5	12.5	25

**Table 4:** Names of the medicinal plants, their families, and extracted parts used in this study.

Names of Medicinal Plant	Family	Extracted Part
*Rota graveolens*	*Rutaceae*	Aerial part (leaves and flowers)
*Camellia sinensis*	*Theaceae*	Leaves
*Olea europaea*	*Oleaceae*	Leaves
*Styrax benzoin*	*Styraceae*	Resin
*Humulus lupulus*	*Cannabaceae*	Female inflorescences (hop cones)
*Ocimum basilicum*	*Lamiaceae*	Aerial part (leaves and flowers)
Propolis (*Apis mellifera*)	*Apidae*	Resin

**Table 5:** List of primer sequences used for quantitative Real-time PCR (qRT-PCR).

Gene	Primer Direction	Sequence 5′-3′	Amplicon Size (bp)	Reference
*5S RNA*	Forward	TGACGATCATAGAGCGTTGG	121	[74]
	Reverse	GATAGGAGCTTGACGATGACCT		
*lasI*	Forward	GTGACGGTAACCACCGTAGG	130	[74]
	Reverse	CTGGGTCTTGGCATTGAGTT		
*lasR*	Forward	CTGTGGATGCTCAAGGACTAC	133	[75]
	Reverse	AACTGGTCTTGCCGATGG		
*rhlI*	Forward	AAGGACGTCTTCGCCTACCT	130	[20]
	Reverse	GCAGGCTGGACCAGAATATC		
*rhlR*	Forward	CATCCGATGCTGATGTCCAACC	101	[20]
	Reverse	ATGATGGCGATTTCCCCGGAAC		

## Supplementary Materials

The following are available online at https://www.mdpi.com/2079-6382/9/9/526/s1, Table S1. Antibiotics breakpoint list. The table is showing different antibiotic breakpoints according to CLSI (2016) guidelines. These breakpoints were used to interpret the results of the antibiotic disc diffusion test to determine the sensitivity of our *P. aeruginosa* isolates to tested antibiotics. Table S2. Qualitative phytochemical analysis and polyphenol contents of ethanol extracts of *Camellia sinensis* and *Olea europaea*. Figure S1. Biofilm formation by *Pseudomonas* isolates on Congo red agar plate. (A): black colonies indicated the EPS production and biofilm formation by *P. aeruginosa*, while (B) red colonies indicated nonbiofilm producers. Congo red agar is a qualitative method performed onto the agar plate surface. Figure S2. *P. aeruginosa* growth with and without the sub-MIC of different herpal extracts. (A) the *P. aeruginosa* (C21) growth curve, while (B) the *P. aeruginosa* (E81) growth curve treated and untreated with with *Camellia sinensis* (3.125 mg/mL), *Olea europaea* (6.25 mg/mL), *Styrax benzoin* (12.5 mg/mL), *Humulus lupulus* (6.25 mg/mL), *Ocimum basilicum* (6.25 mg/mL), Propolis (6.25 mg /mL), and Ruta graveolans (12.5 mg/mL) with respect to the control every 2 h at 37 °C over 24 h. The data represent the mean value ± standard deviation and experiments performed in triplicate.
