# Supplementary material for: Olive Leaf Extract Modulates Quorum Sensing Genes and Biofilm Formation in Multi-Drug Resistant Pseudomonas aeruginosa
Source: Antibiotics (Basel). 2020 Aug 19;9(9):526. doi: 10.3390/antibiotics9090526 (PMC7560099; doi:10.3390/antibiotics9090526)
Supplement: Supplementary file 1 [file antibiotics-09-00526-s001.pdf]

Supplementary materials

Type of the Paper (Article)

Olive leaf extract modulates quorum sensing genes and biofilm formation in multi-drug resistant *Pseudomonas aeruginosa*

Nazly R. El-sayed[1], Reham Samir [2]*, Lina J. M. Abdel-Hafez[1], and Mohammed A. Ramadan[2]

[1] Department of Microbiology and Immunology, Faculty of Pharmacy, 6 OctoberUniversity, 12585, Giza, Egypt

[2] Department of Microbiology and Immunology, Faculty of Pharmacy, Cairo University, 11562, Cairo, Egypt

.

*Correspondence: email address; reham.samer@pharma.cu.edu.eg; Tel. :(02-01224172986)

Supplementary table 1

Table S1. Antibiotics breakpoints list. The table is showing different antibiotics breakpoints according to CLSI (2016) guidelines. These breakpoints were used to interpret the results of antibiotics disc diffusion test to determine the sensitivity of our *P. aeruginosa* isolates to tested antibiotics.

Antimicrobial agent	Disc potency	Zone diameter (mm)		
		Susceptible	Intermediate	Resistant
Amikacin	30 µg	≥17	15-16	≤14
Aztreonam	30 µg	≥22	16-21	≤15
Cefepime	30 µg	≥18	15-17	≤14
Ceftazidime	30 µg	≥18	15-17	≤14
Ciprofloxacin	5 µg	≥21	16-20	≤15
Colistin	10 µg	≥11	-	≤10
Gentamicin	10 µg	≥15	13-14	≤12
Imipenem	10 µg	≥19	16-18	≤15
Levofloxacin	5 µg	≥17	14-16	≤13
Meropenem	10 µg	≥19	16-18	≤15
Piperacillin/Tazobactam	10/100 µg	≥21	15-20	≤14
Ticarcillin/Clavulanic acid	10/75 µg	≥24	16-23	≤15
Tobramycin	10 µg	≥15	13-14	≤12

Table S2. Qualitative phytochemical analysis and polyphenol contents of ethanol extracts of *Camellia sinensis* and *Olea europaea*

**Plant constituent**	***Camellia sinensis***	***Olea europaea***
Tannin	+	+
Saponins	+	-
Anthraquinones	-	-
Alkaloid	+	+
Flavonoids	+	+
Phenols	+	+
Cardiac glycoside	-	-
Glycoside	+	+
Terpenoids	+	+
Anthocyanin	+	+
Polyphenol contents (mg/kg)	2.93	2.07

+ = Presence, - = Absence

Supplementary figure S1:

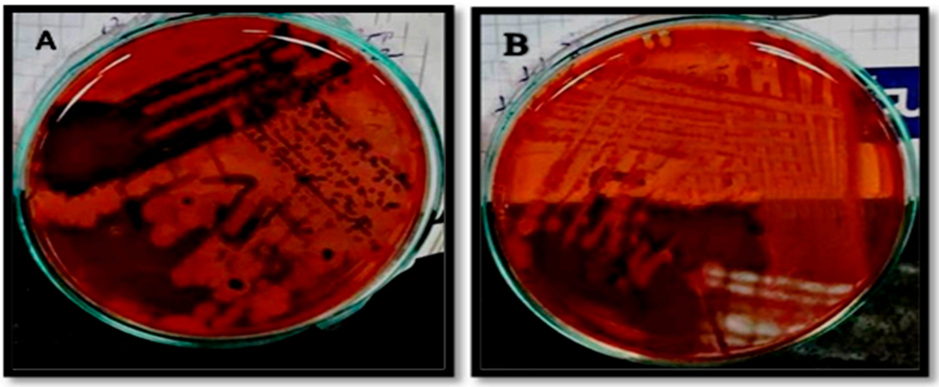

Figure S1. Biofilm formation by *Pseudomonas* isolates on Congo red agar plate. (A) Black colonies indicated the EPS production & biofilm formation by *P. aeruginosa* while (B) red colonies indicated non-biofilm producers. Congo red agar is a qualitative method performed onto agar plate surface.

Supplementary figure S2:

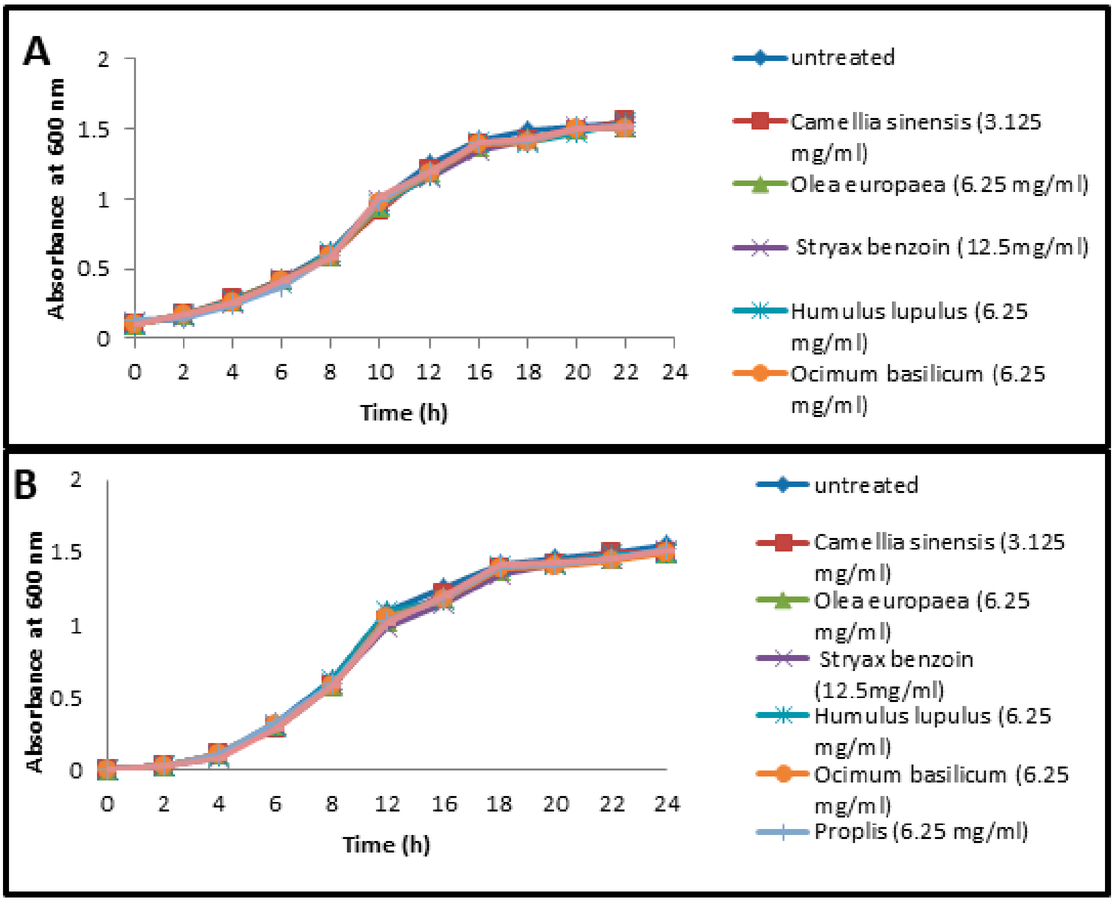

Figure S2. *P. aeruginosa* growth with and without the sub-MIC of different herpal extracts. (A) *P. aeruginosa* PAO1 (C21) growth curve, while (B) *P. aeruginosa* PAO1 (E81) growth curve treated and untreated with with *Camellia sinensis* (3.125 mg/ml), *Olea europaea* (6.25 mg/ml), *Styrax benzoin* (12.5mg/ml), *Humulus lupulus* (6.25 mg/ml), *Ocimum basilicum* (6.25 mg/ml), Propolis (6.25 mg /ml), Ruta graveolans (12.5 mg/ml) with respect to control every 2h at 37°C over 24 h.. The data represent as mean value ± standard deviation and experiments performed in triplicate.
